# Integrative proteomic and metabolomic analysis reveals that ouabain inhibits DLBCL proliferation by reprogramming arginine-driven metabolic networks

**DOI:** 10.3389/fphar.2026.1929830

**Published:** 2026-09-21

**Authors:** Yuan Wei, Yanhuan Yu, Yuning Zhu, Xinwen Zhang, Zhuoling Zhao, Sinan Wang, Fei Fei, Bei Cao, Runbin Sun, Juan Li

**Affiliations:** 1 Department of Phase I Clinical Trials Unit, Nanjing Drum Tower Hospital Clinical College of Nanjing University of Chinese Medicine, Nanjing, China; 2 Department of Phase I Clinical Trials Unit, Nanjing Drum Tower Hospital, China Pharmaceutical University, Nanjing, China; 3 Department of Phase I Clinical Trials Unit, Nanjing Drum Tower Hospital, Affiliated Hospital of Medical School, Nanjing University, Nanjing, China

**Keywords:** DLBCL, integrated analysis, metabolomics, ouabain, proteomics

## Abstract

Diffuse large B-cell lymphoma (DLBCL) is the most common subtype of non-Hodgkin lymphoma (NHL), with an increasing incidence in recent years. Ouabain, a cardiotonic steroid, has demonstrated anti-tumor effects in various cancers including DLBCL, but its mechanism of action is not yet fully understood. We first used liquid chromatography-tandem mass spectrometry (LC-MS) for proteomics and metabolomics to analyze changes in DLBCL cells with ouabain administration. The results revealed significant alterations in the abundance of 303 proteins and 206 metabolites between the ouabain-treated and control groups. Proteins like MYC, RAD51, AURKB, CENPF, and RRM2 were downregulated, along with metabolism-related proteins ARG2, OAT, and TYMS. Metabolite levels including glutamate, glutathione, ornithine, and taurine were significantly altered. The integrated analysis revealed that arginine metabolism serves as a central hub, coordinating changes in glutathione metabolism and nucleotide biosynthesis, contributing to increased oxidative stress, replication stress, and growth inhibition. These findings suggest that ouabain disrupts arginine-driven metabolic network to alter redox balance and inhibit tumor cell proliferation, providing a promising therapeutic rationale for targeting metabolic vulnerabilities in DLBCL.

## Graphical Abstract

**Figure:**
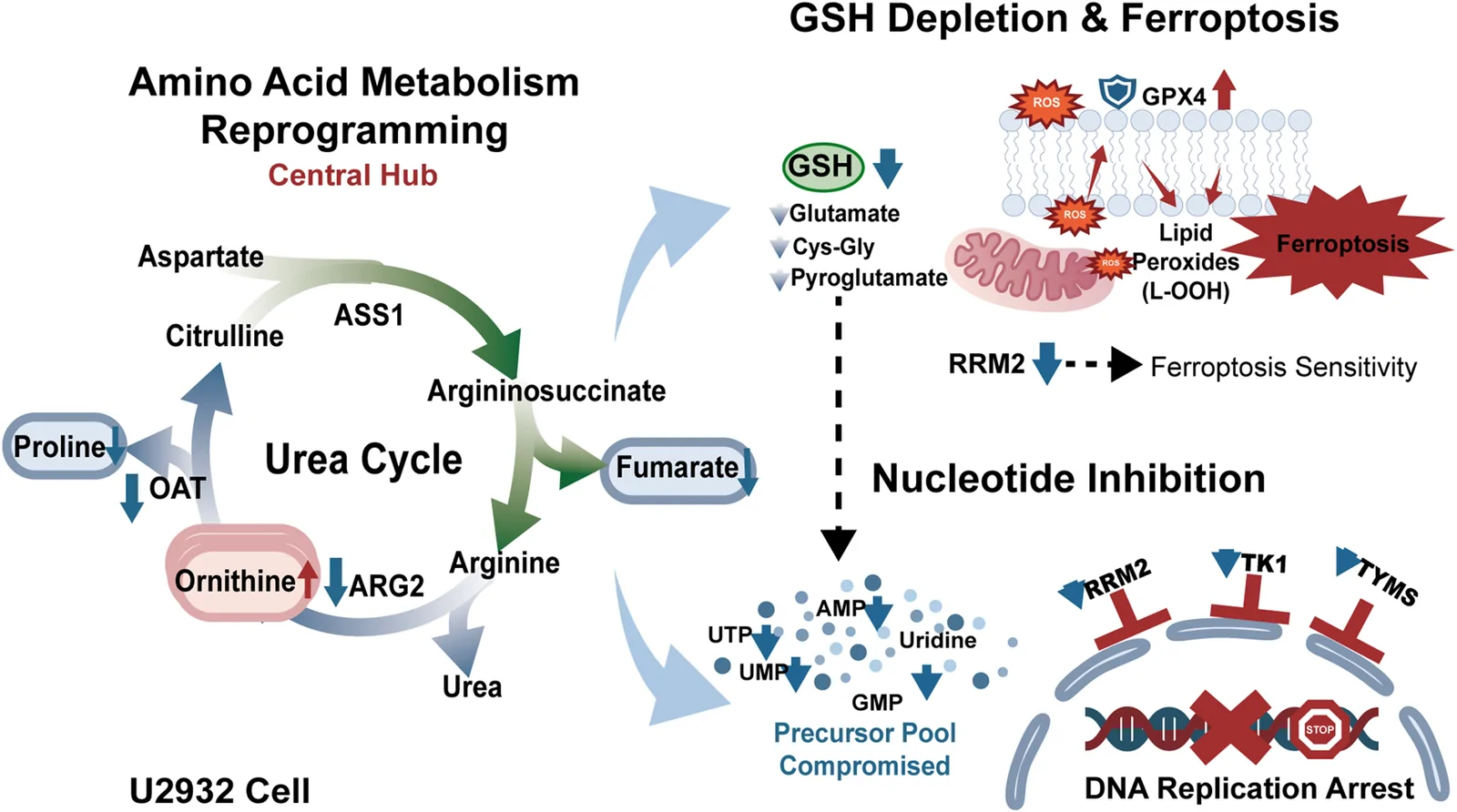

## 1 Introduction

Diffuse large B-cell lymphoma (DLBCL) is the most common subtype of non-Hodgkin lymphoma (NHL) and an aggressive hematological malignancy characterized by marked invasiveness and pronounced biological heterogeneity (Huang et al., 2020). Globally, DLBCL accounts for approximately 30%–40% of all NHL cases, with incidence increasing with age and a higher prevalence in males (Chen et al., 2013; Wang, 2023). Based on gene expression profiling, DLBCL is primarily classified into activated B-cell-like (ABC) and germinal center B-cell-like (GCB) subtypes, although 10%–15% of cases remain unclassifiable (Alizadeh et al., 2000). Despite the widespread use of the R-CHOP regimen (rituximab, cyclophosphamide, doxorubicin, vincristine, and prednisone), which achieves long-term remission in 60%–70% of patients, approximately 30%–40% develop relapsed or refractory disease with a median survival of less than 2 years (Crump et al., 2017; Liu and Barta, 2019; Ejtehadifar et al., 2025). These limitations underscore the urgent need to explore novel therapeutic strategies.

Ouabain, a kind of cardiac glycoside, is a classical inhibitor of Na^+^/K^+^-ATPase enzyme and has long been used in the treatment of heart disease (Li et al., 2010). In recent years, increasing evidence has demonstrated that ouabain exerts potent anti-tumor effects in multiple malignancies, including lung cancer (Chanvorachote and Pongrakhananon, 2013), acute myeloid leukemia (Ihenetu et al., 2007; Tailler et al., 2012), liver cancer (Ozdemir et al., 2012), melanoma (Prassas and Diamandis, 2008) and DLBCL(Zheng et al., 2021). Mechanistically, ouabain has been shown to induce cell cycle arrest and apoptosis, elevate intracellular reactive oxygen species (ROS), suppress oncogenic transcriptional programs, and impair tumor cell migration and invasion (Wang et al., 2009; Wang et al., 2015; Patel, 2016). However, the precise molecular mechanisms underlying its anti-tumor activity in DLBCL remain incompletely understood.

Metabolic reprogramming is a hallmark of cancer and plays a critical role in sustaining rapid proliferation and survival (Hong et al., 2024). Studies have also shown that abnormal metabolic phenotypes in DLBCL patients (Zhang et al., 2025a), such as elevated lactate dehydrogenase and increased glutamine consumption, are closely associated with tumor stage, aggressiveness, and clinical prognosis. Further studies have revealed that DLBCL cells undergo remarkable remodeling of amino acid metabolic profiles: tumor cells significantly enhance the uptake of key amino acids including glutamine, serine and leucine by highly expressing amino acid transporters such as LAT1 and ASCT2 (Cormerais et al., 2018; Perucha et al., 2025). Meanwhile, the glutamine catabolic pathway is persistently activated and the serine-glycine-one-carbon unit metabolic pathway is markedly upregulated, thereby meeting the material and energy requirements for tumor cell proliferation (Ducker et al., 2017). Recent studies have indicated that the dysregulation of amino acid availability and metabolic patterns can profoundly affect tumor cells by regulating key biological processes including glutathione synthesis, oxidative stress response, and DNA replication capacity (Chen et al., 2021). For instance, glutamate derived from glutamine metabolism serves as a core substrate for glutathione synthesis, while one-carbon units generated from serine catabolism directly participate in nucleotide synthesis (Ducker et al., 2017). Together, they form the metabolic basis for maintaining redox homeostasis and safeguarding genomic stability in DLBCL cells. Nevertheless, how ouabain reshapes the amino acid metabolic network of DLBCL cells and how metabolic remodeling coordinately regulates cellular redox homeostasis and nucleotide metabolism have not been systematically investigated.

With the rapid development of high-throughput omics technologies, proteomics and metabolomics have become powerful tools for dissecting cancer-associated metabolic reprogramming (Chi et al., 2025; Malhotra et al., 2025; Zelkowska et al., 2025). Integrated proteomic and metabolomic analyses enable the simultaneous characterization of metabolic enzymes and their corresponding metabolites, providing a comprehensive view of pathway-level regulation (Hoffman et al., 2017). In DLBCL, metabolomic studies have revealed that glutathione depletion and lipid peroxidation-induced ferroptosis contribute to tumor growth inhibition (Zhang et al., 2019; Zhou et al., 2023), while proteomic analyses have identified key metabolic regulators such as HK2(Zhang et al., 2025b)and GLS1 (Gomez Solsona et al., 2023). However, there is still no research that systematically explores the coordinated protein-metabolite regulatory landscape underlying ouabain-induced metabolic reprogramming in DLBCL, particularly within interconnected amino acid pathways.

In this study, we employed an integrated proteomics and metabolomics approach to investigate the metabolic and proteomic alterations induced by ouabain in U2932 cells, an activated B-cell-like (ABC) DLBCL model. By delineating this proteomics and metabolic network, our study aimes to provide a mechanistic insight into the anti-tumor effects of ouabain and highlights potential metabolic vulnerabilities that may be exploited for combination therapeutic strategies in DLBCL.

## 2 Materials and methods

### 2.1 Cell culture and reagents

The human-derived cell line U2932 was cultured in RPMI 1640 medium supplemented with 2 mM L-glutamine, 10% fetal bovine serum, 100 μg/mL penicillin, and 100 μg/mL streptomycin (all from Gibco, Carlsbad, California, USA) in a humidified atmosphere at 37 °C with 5% CO_2_. Ouabain octahydrate (≥95% HPLC, powder) was purchased from Sigma-Aldrich (St. Louis, Missouri, USA).

### 2.2 Cell viability

The CCK-8 assay, based on cellular metabolic activity, was used to evaluate cell proliferation. U2932 cells (3 × 10^4^/mL) were seeded in 96-well plates and incubated with ouabain (25, 50, 100, 150, 200 and 250 nM) or a solvent control (0 nM, containing 0.1% DMSO) for 24 h. Cell viability was measured by adding CCK-8 reagent (1:10 dilution, Servicebio, Wuhan, China) and incubating for 3 h at 37 °C. Absorbance at 450 nm was measured by a microplate reader (TECAN Spark, Switzerland).

### 2.3 Proteomics analysis

U2932 cells (5 × 10^6^/mL) were seeded in 6-well plates and incubated for 24 h with either 0 nM (solvent control, containing 0.1% DMSO, designated as the CON group) or 118 nM ouabain (IC50, designated as the OUA group). After incubation, cells were collected by centrifugation (800 × g, 5 min), washed once with 1 mL of physiological saline, and resuspended in 100 μL of ultrapure water (Milli-Q system, Millipore, Bedford, USA). Samples were stored at −80 °C until analysis.

Proteomic analysis was performed by Suzhou PANOMIX Biomedical Tech Co., Ltd (Suzhou, Jiangsu, China). Briefly, intracellular proteins were extracted, and the lysates were centrifuged at 12,000 x g for 5 min at 4 °C. The resulting supernatant was diluted 1:1 with water, and protein concentration was measured by NanoDrop 2000 spectrophotometer (Thermo Fisher Scientific, Waltham, USA). Subsequently, the protein samples underwent reduction, alkylation, and enzymatic digestion with trypsin at a ratio of 1:50 overnight at 37 °C. Digested peptides were reconstituted and loaded onto a liquid chromatography system equipped with a reversed-phase analytical column (25 cm × 100 μm i. d). The mobile phase consisted of solvent A (0.1% formic acid in water) and solvent B (acetonitrile). A gradient elution was applied (0–25 min, 5%–30% B; 25–30 min, 30%–100% B) at a flow rate of 300 nL/min. Quantitative proteomic profiling was performed in data-independent acquisition (DIA) mode on a Thermo Scientific Orbitrap Astral mass spectrometer. The parameters were configured as follows: MS1 resolution at 120,000 with a scan range of 350–1,500 m/z; DIA MS2 resolution at 30,000, and higher-energy collisional dissociation (HCD) normalized collision energy (NCE) at 27%. The raw DIA data were processed and searched using DIA-NN (version 1.8). Gene Ontology (GO) and Kyoto Encyclopedia of Genes and Genomes (KEGG) pathway enrichment analyses were performed on the differentially expressed proteins. Protein-protein interaction (PPI) networks were constructed based on the STRING database (V12.0) and visualized using GraphPad Prism software (V9.0, Boston, USA). The raw mass spectrometry proteomics datasets have been deposited to the iProX partner repository with the dataset identifier PXD073963.

### 2.4 Untargeted metabolomics analysis

Cell collection for metabolomics samples followed the same procedure as described above. Samples were stored at −80 °C. For metabolite extraction, cell samples were subjected to three freeze-thaw cycles to facilitate cell lysis. Then, 300 μL of methanol containing the internal standard (IS, 1 μg/mL 4-Chlorophenylalanine) was added. The mixture was transferred to Eppendorf tubes, vortexed for 3 min, and centrifuged at 18,000 xg for 10 min at 4 °C. A 90 μL aliquot of the supernatant was transferred into a glass vial for analysis. Quality control (QC) samples were prepared by pooling equal volumes of the supernatant from each sample.

Metabolic profiling was performed using a liquid chromatography system coupled with a quadrupole time-of-flight tandem mass spectrometer (AB SCIEX TripleTOF 5600 LC-QTOF/MS, Foster City, USA) equipped with a Waters HSS T3 column (2.1 × 100 mm, 1.8 μm). The flow rate was 0.3 mL/min, the column temperature was set to 40 °C, and the injection volume was 5 μL. The mobile phase consisted of 0.1% formic acid in water (phase A) and acetonitrile (phase B) with a gradient elution: 0–1.5 min, 10% B; 1.5–9 min, 10%–90% B; 9–12 min, 90% B; 12–12.1 min, 90%–10% B; and 12.1–15 min, 10% B. Mass detection was conducted in both positive (5500 V) and negative (−4500 V) ion modes using Turbo V electrospray ionization (ESI). The key source parameters were as follows: ion source gas 1 (GS1), 60 psi; ion source gas 2 (GS2), 60 psi; curtain gas (CUR), 35 psi; declustering potential (DP), 60 V; collision energy (CE), 35 eV; source temperature (TEM), 550 °C. The mass scan range was set to 50–1,200 m/z. Raw data were converted to mzXML format using MSconvert software (V3.0). Metabolite identification and KEGG (Kanehisa and Goto, 2000) database matching were performed using R Project (V4.4.1). Further data analysis and visualization were executed using an in-house developed R package.

### 2.5 Targeted metabolomics analysis

Chemicals and Reagents: Amino acid standards and IS (4-chlorophenylalanine) were purchased from Sigma-Aldrich and Aladdin Co., Ltd (Shanghai, China). Formic acid and other basic LC-MS grade solvents were obtained from Merck KGaA (Darmstadt, Germany).

Preparation of Calibration Standards and Sample Extraction:Standard stock solutions of amino acids were prepared in methanol at a concentration of 10 mM and subsequently diluted to 500 μM. To construct the calibration curves, a mixed standard working solution was serially diluted to yield 11 concentration levels: 0.01, 0.02, 0.05, 0.1, 0.2, 0.5, 1, 2, 5, 10, and 20 μM. The IS stock solution was prepared at a concentration of 1 μg/mL.

For the extraction of intracellular amino acids (n = 3 for each group), the procedure was consistent with the untargeted metabolomics analysis. Following cell collection, a 30 μL aliquot of the sample was mixed with 120 μL of methanol containing the IS for protein precipitation. The mixture was vortexed for 3 min and centrifuged at 18,000 xg for 10 min at 4 °C. A 100 μL aliquot of the supernatant was carefully collected and transferred into a glass vial for direct LC-MS/MS analysis to determine the changes in amino acids before and after ouabain administration.

Targeted quantification was performed using an ultra-performance liquid chromatography system coupled with a 4,500 mass spectrometer (AB SCIEX, Toronto, Canada). The mobile phase composition was identical to that used in the untargeted analysis, with a gradient elution: 0–1.5 min, 60% B; 1.5–7 min, 60%–95% B; 7–10 min, 95% B; 10–10.1 min, 95%–60% B; and 10.1–12.5 min, 60% B. The mass spectrometer operated in multiple reaction monitoring (MRM) mode with the following source parameters: CUR 35 psi, collision gas (CAD) eight psi, GS1 35psi, GS2 55 psi, and TEM 360 °C. The optimum MRM transitions for the amino acids and the IS are detailed in Supplementary Table S4. Finally, Analyst® software (V1.7.1) was utilized for peak identification, and absolute concentrations were calculated based on the standard curves and IS concentrations.

### 2.6 Quantitative PCR

Total mRNA was extracted using MolPure® Cell/Tissue Total RNA Kit (Yeasen, Shanghai, China). The RNA was reverse transcribed into complementary DNA using a reverse transcription kit (Vazyme, Nanjing, China). Quantitative PCR was performed using SYBR Green reagent (Vazyme, Nanjing, China) with β-actin as the internal control. QPCR was performed using the Roche LightCycler® 480 II System (Roche Diagnostics, Mannheim, Germany). The primer sequences used were listed in Supplementary Table S2.

### 2.7 Statistical analysis

For metabolomic and proteomic analyses, relative quantification based on peak areas was subjected to median normalization and subsequent Log2 transformation to meet normality assumptions for statistical analyses. All experiments were performed in triplicate and were repeated at least three times (n ≥ 3). Results are presented as mean ± SD. Statistical significance was determined using the unpaired, two-tailed Student’s t-test using GraphPad Prism. Significant differences for omics data were considered based on a nominal p-value <0.05 and a fold-change (FC) > 1.5 or <0.67. To account for multiple testing, the false discovery rate (FDR) was calculated using the Benjamini–Hochberg method. Given the exploratory nature of the omics analysis and the sample size, the nominal p-value <0.05 was retained as the primary significance threshold to prevent excessive false negatives and preserve biologically relevant network alterations. The calculated q-values are provided in the Supplementary Datasets for comprehensive reference.

## 3 Results

### 3.1 Ouabain induces widespread proteomic alterations in U2932 cells

To verify the anti-proliferative and pro-apoptotic effects of low-concentration ouabain on DLBCL cells reported in previous research (Hong et al., 2024), we first used the CCK-8 assay to determine its half-maximal inhibitory concentration (IC50) (Supplementary Figure S1). We then utilized LC-MS/MS to analyze the cellular proteomes of the CON group and the OUA group. Principal component analysis (PCA) was performed on all samples, showing tight clustering, which confirmed the reproducibility of experimental procedures and the stability of the instrument (Figure 1A). After data filtering, we identified a total of 7,893 proteins by comparing the acquired mass spectrometry data with a protein sequence database (Figure 1B and Supplementary Data 2). A threshold for significant upregulation was set at FC > 1.5 (p < 0.05), and for downregulation, FC < 0.67 (p < 0.05).

**FIGURE 1:**
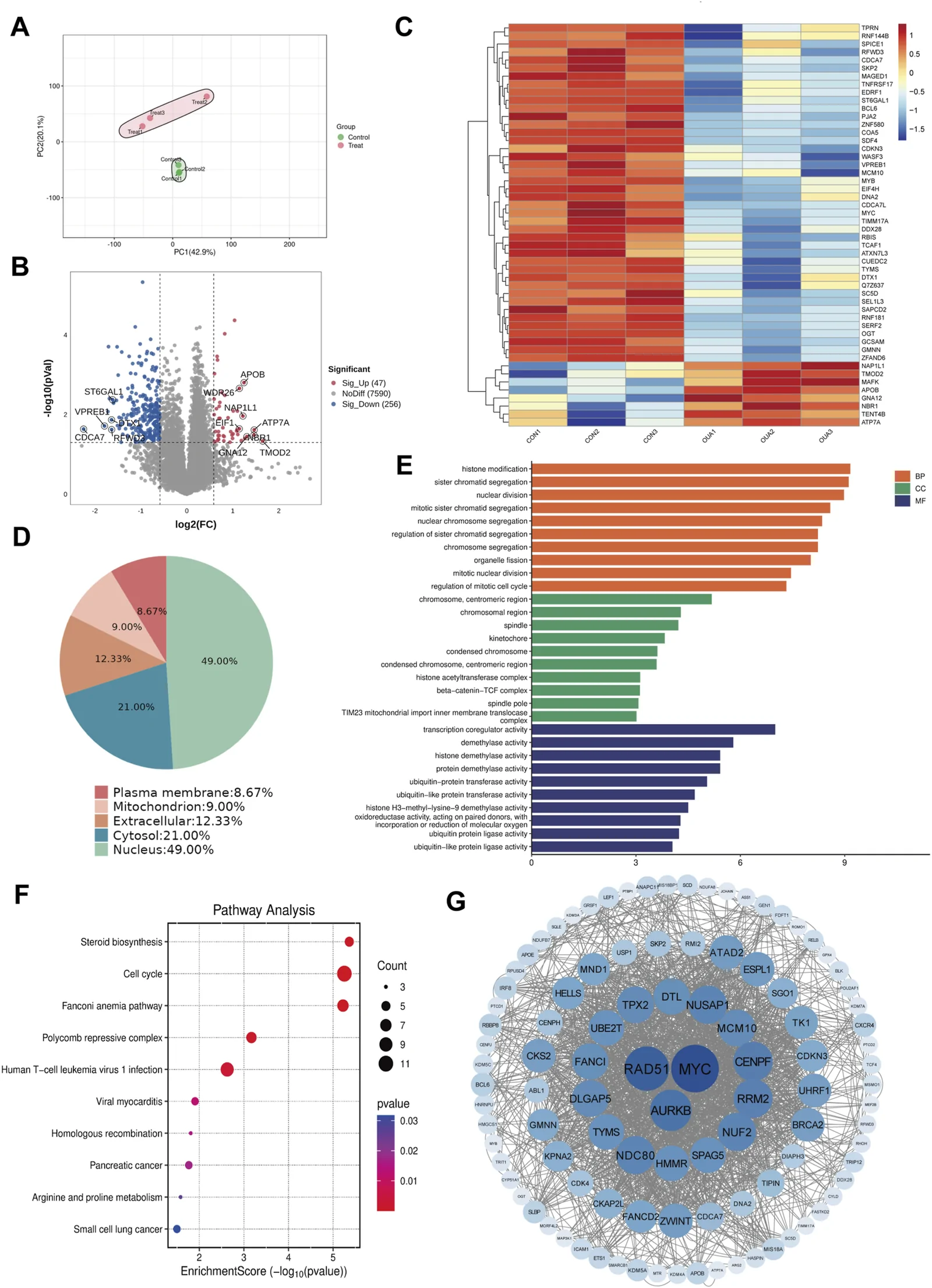
Proteomic analyses of the Ouabain and CON groups (n = 3). **(A)** PCA plot of the OUA and CON groups. **(B)** Volcano plot of the identified proteins. Red dots and blue dots represent significantly upregulated and downregulated proteins, respectively. **(C)** Hierarchical clustering heatmap of the differentially expressed proteins (DEPs). Red indicates high expression, and blue indicates low expression. **(D)** Subcellular localization of the identified DEPs. **(E)** GO functional classification of the DEPs. The horizontal axis represents the enrichment score, and the vertical axis represents the secondary functional classification within the primary GO categories (Biological process/Cellular component/Molecular function) **(F)**. KEGG pathway enrichment analysis of the DEPs. The dot plot shows the 10 most significantly enriched pathways. **(G)** PPI network of the DEPs between the OUA and CON groups based on the STRING database.

As a result, 303 differentially expressed proteins (DEPs) were found in the OUA group compared to the CON group, with 47 upregulated and 256 downregulated (Supplementary Figure S2). A heatmap showing the relative expression of DEPs in both groups is presented in Figure 1C. Subcellular localization analysis indicated that the largest proportion of DEPs were localized to the nucleus (Figure 1D), suggesting that ouabain has a significant impact on intranuclear processes.

GO enrichment analysis (Figure 1E) and KEGG enrichment analysis (Figure 1F) were conducted on the DEPs. A total of 654 GO terms were identified as statistically significant. Among the enriched Cellular Component categories, the majority were related to structures involved in cell division. In the Biological Process category, most terms were related to chromosome segregation and cell division. The enriched terms in the Molecular Function category focused primarily on demethylation, ubiquitination, and redox activity. KEGG analysis revealed that the enriched pathways were primarily associated with cell proliferation, DNA damage repair, metabolic regulation, and tumorigenesis. These findings are consistent with previous reports indicating that cardiotonic steroids can inhibit tumor cell division and induce apoptosis (López-Lázaro, 2007; Prassas and Diamandis, 2008).

We constructed a PPI network and screened for core nodes using the Maximal Clique Centrality (MCC), Maximum Neighborhood Component (MNC), and Degree algorithms (Figure 1G). This analysis identified six hub genes, including myelocytomatosis oncogene (MYC), DNA repair protein RAD51 homolog 1 (RAD51), Aurora kinase B (AURKB), centromere protein F (CENPF), Thymidylate synthase (TYMS) and Ribonucleoside-diphosphate reductase subunit M2 (RRM2) (Figure 1G), which are critically involved in key biological pathways such as cell cycle regulation, DNA damage repair, mitotic spindle assembly, and chromosome segregation.

Based on the proteomics data, the expression of key proteins related to cell proliferation was significantly suppressed, while the expression of some proteins related to immune recognition and stress response was upregulated (Supplementary Figure S3). The expression of MYC(FC = 0.38) (Duffy et al., 2021), an oncoprotein that globally regulates transcription and metabolism, showed significant downregulation. B cell lymphoma 6 (BCL6) (FC = 0.42) (McPhail et al., 2018), a key driver gene in B-cell non-Hodgkin lymphoma, was also significantly decreased. In conclusion, ouabain may exert a potent anti-tumor cell proliferation effect through multi-target and multi-pathway synergistic actions.

### 3.2 Metabolic profiling reveals distinct metabolite perturbations in ouabain-treated U2932 cells

Given the potential role of metabolites such as glucose (Segel et al., 1978), lipids (Wang et al., 2024), and amino acids (Zheng et al., 2021) in regulating DLBCL, we performed LC-TOF/MS metabolomics analysis to investigate changes in U2932 cells after ouabain treatment. Before conducting a detailed analysis of specific metabolic alterations, we applied the OPLS-DA model to evaluate metabolic differences between the OUA and CON groups (Figure 2A). The OPLS-DA model permutation tests revealed clear separation between the OUA and CON groups in both electrospray ionization positive (ESI+) and negative (ESI-) modes, indicating reproducible chemical differences. The permutation test results also confirmed that the model was not overfitted (R^2^X = 0.471, R^2^Y = 1, and Q^2^ = 0.687; Supplementary Figure S4).

**FIGURE 2:**
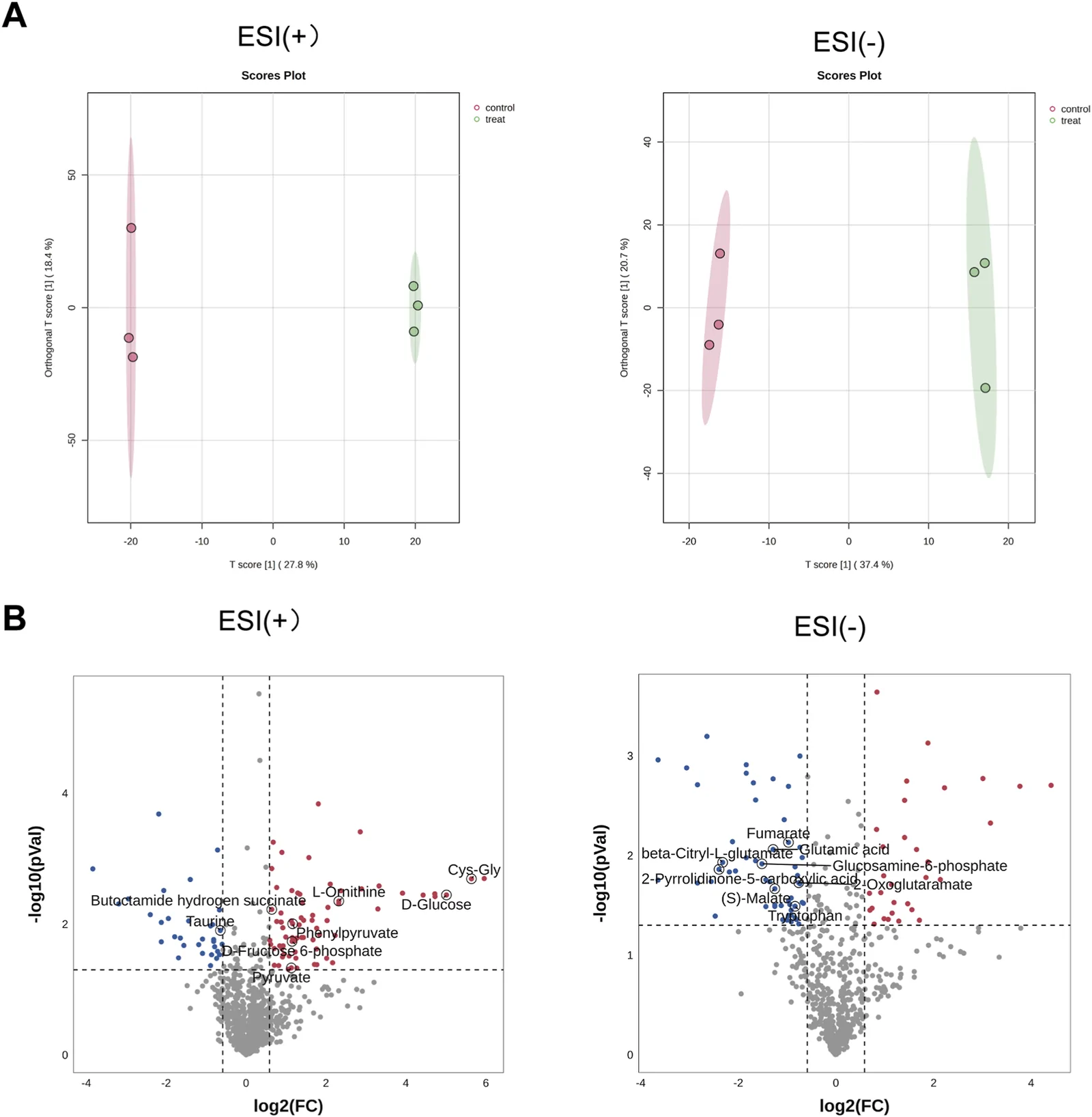
Metabolite changes in ESI (+) and ESI (−) modes (n = 3). **(A)** OPLS-DA model scores plots for the OUA and CON groups in ESI (+) and ESI (−) modes. **(B)** Volcano plots showing the DAMs. The screening criteria including p < 0.05, VIP> 1, and FC > 1.5 or FC < 0.67. The red and blue dots represent significantly increased and decreased metabolites, respectively.

Following this preliminary analysis, we employed univariate and multivariate statistical methods to identify differentially abundant metabolites (DAMs) in U2932 cells.

After ouabain treatment. Based on the screening criteria (FC > 1.5 or <0.67, VIP >1, p < 0.05), 206 DAM were identified, with 97 upregulated and 109 downregulated (Supplementary Figure S5 and Supplementary Data 3), and the volcano plot for all the metabolites was shown in Figure 2B. The DAMs were classified into 16 chemical classes, and the distribution of metabolites in each class is shown in Figure 3A. Organic acids and derivatives, as well as nucleosides, nucleotides, and analogues, accounted for the highest proportion (18.45%), followed by lipids and lipid-like molecules (12.62%).

**FIGURE 3:**
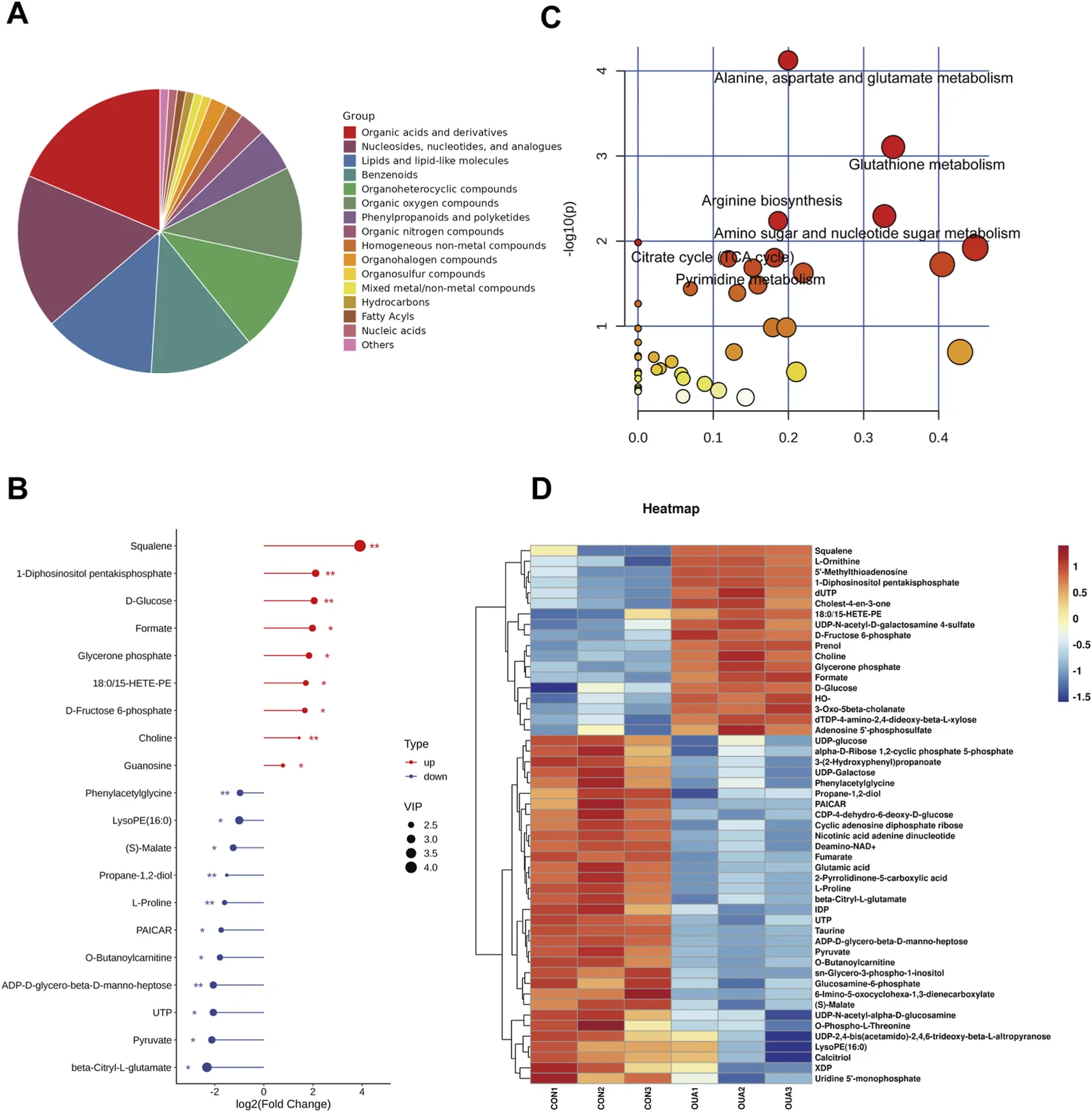
Metabolomic analysis of the OUA group and the CON group (n = 3). **(A)** Pie chart showing the functional classification of the DAMs. **(B)** Lollipop plot illustrating the top 10 upregulated and downregulated DAMs. The horizontal axis represents the logarithmically transformed fold changes ( $log_{2}\left(FC\right)$ ). The node size corresponds to the VIP values, and the color indicates the regulation type (red for upregulated, blue for downregulated). **(C)** Bubble plot of KEGG pathway enrichment analysis for the DAMs. **(D)** Hierarchical clustering heatmap of the selected DAMs between the OUA and CON groups.

In addition to proteomics, metabolomics provided a deeper understanding of how ouabain affects metabolic processes within U2932 cells. The top 20 most significantly changed metabolites are displayed in Figure 3B, predominantly in lipid metabolism (Squalene), energy metabolism (D-Fructose-6-phosphate, Pyruvate), amino acid derivative metabolism (Taurine), as well as nucleotide and signal metabolism pathways. The top five upregulated metabolites were Squalene, 3-Oxo-5beta-cholanate, 1-Diphosinositol pentakisphosphate (1-PP-IP5), D-Glucose and Formate, and the top five downregulated metabolites were Taurine, UDP-glucose, CDP-4-dehydro-6-deoxy-D-glucose (Digitalose), 2-Pyrrolidinone-5-carboxylic acid, and beta-Citryl-L-glutamate.

To identify the relevant metabolic pathways, KEGG enrichment analysis was performed on the differentially abundant metabolites (Figure 3C). According to KEGG analysis, 41 metabolic pathways were classified and annotated. Based on the enrichment significance, the top 10 significantly enriched KEGG pathways were amino acid metabolism pathways, including Alanine, aspartate and glutamate metabolism, Arginine and proline metabolism, and Phenylalanine metabolism, energy metabolism pathways such as the Citrate cycle (TCA cycle), Glycolysis/Gluconeogenesis and Pyruvate metabolism, nucleotide metabolism pathways including Pyrimidine metabolism and Purine metabolism, as well as lipid and oxidative stress-related metabolic pathways. The corresponding heatmap is shown in Figure 3D. Among these, Alanine, aspartate and glutamate metabolism (p = 9.07E-05) and Glutathione metabolism (p = 9.13E-04) were the most significantly enriched. The former is involved in the interconversion of amino acids and nitrogen metabolism, linking carbohydrate and amino acid metabolism to regulate intracellular nitrogen balance and energy supply. The latter participates in cellular damage repair and stress adaptation through antioxidant activity, detoxification, and maintenance of cellular redox homeostasis. Additionally, pyrimidine metabolism (p = 0.00423) and arginine biosynthesis (p = 0.00637) were also significantly enriched, which are related to DNA/RNA synthesis and cell proliferation, arginine synthesis and immune regulation, respectively. Furthermore, energy metabolism pathways including the TCA cycle, Glycolysis/Gluconeogenesis and Pyruvate metabolism were all markedly enriched.

### 3.3 Integrated analyses of proteomics and metabolomics reveal ouabain significantly changed the arginine metabolism in U2932 cells

To systematically investigate the mechanisms underlying ouabain’s effects in U2932 cells, we performed an integrated proteomics and metabolomics analysis. KEGG pathway enrichment analysis of DEPs and DAMs identified 14 overlapping pathways (Figures 4A,B). Based on p < 0.05 and biological relevance (Supplementary Table S3), we focused on five core pathways: Alanine, aspartate and glutamate metabolism, Glutathione metabolism, Arginine biosynthesis, Arginine and proline metabolism, and Pyrimidine metabolism. These integrated results suggest that ouabain exerts its anti-tumor effects by disrupting multiple key metabolic pathways in a coordinated manner.

**FIGURE 4:**
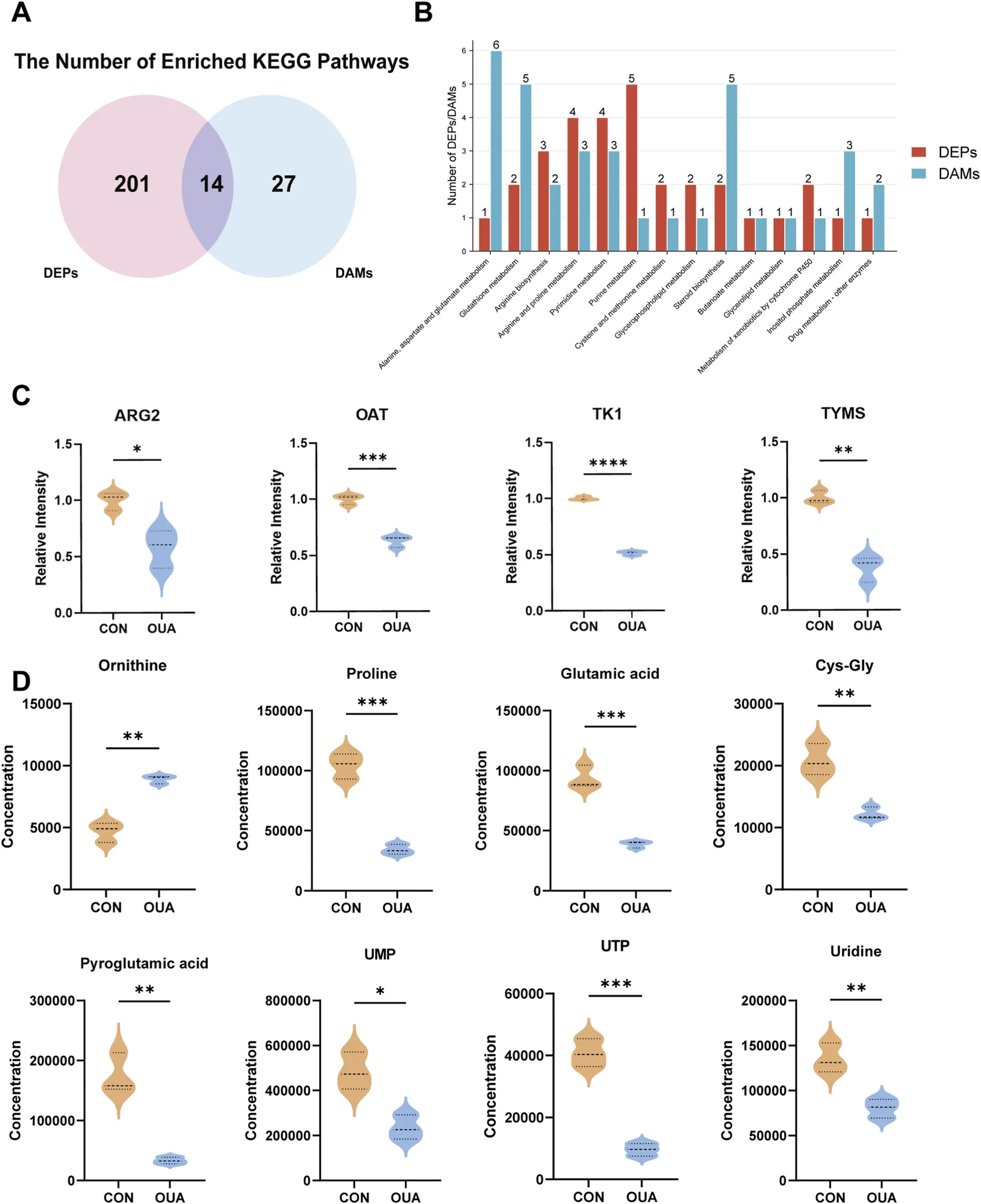
Integrated analyses of proteomics and metabolomics. **(A)** Venn diagram showing the overlap of significantly enriched KEGG pathways between the proteomic (DEPs, pink) and metabolomic (DAMs, blue) analyses. **(B)** Bar plot detailing the top intersecting KEGG pathways enriched by both DEPs (red bars) and DAMs (blue bars). **(C)** Violin plots showing the relative intensities of key DEPs involved in the intersecting pathways. **(D)** Violin plots showing the relative intensities of key DAMs involved in the intersecting pathways (*p < 0.05, **p < 0.01, ***p < 0.001, ****p < 0.0001).

Integrated pathway mapping revealed that these pathways are metabolically interconnected, particularly in terms of amino acid utilization, redox regulation, and nucleotide biosynthesis. This interconnectedness indicates that ouabain induces a complex reprogramming of metabolic networks, leading to cumulative metabolic stress and growth inhibition. A schematic overview of these integrated metabolic alterations is presented in Figures 4C,D.

The integrated analysis revealed significant reprogramming of the arginine-centered amino acid metabolic network in U2932 cells following ouabain treatment (Figure 5A). Ouabain treatment upregulated argininosuccinate synthase 1 (ASS1), driving the metabolic flux from aspartate and citrulline toward argininosuccinate (Supplementary Figure S3 B1). In contrast, arginase 2 (ARG2) expression was markedly reduced, limiting the conversion of arginine into ornithine and urea (Figure 3C). As a result, intracellular ornithine levels increased, further influencing metabolic pathways linked to amino acid and nucleotide metabolism (Figure 4D). Simultaneously, downregulation of ornithine δ-aminotransferase (OAT) impaired the conversion of ornithine to proline, leading to a significant decrease in proline levels. This shift in arginine metabolism creates a state characterized by increased flux toward arginine synthesis while limiting the use of its downstream metabolites like proline. Importantly, this reprogramming links arginine metabolism to the regulation of redox balance and nucleotide synthesis (Liang et al., 2018; Phang, 2019; Mossmann et al., 2023; Xia et al., 2025), thereby orchestrating changes in multiple cellular processes.

**FIGURE 5:**
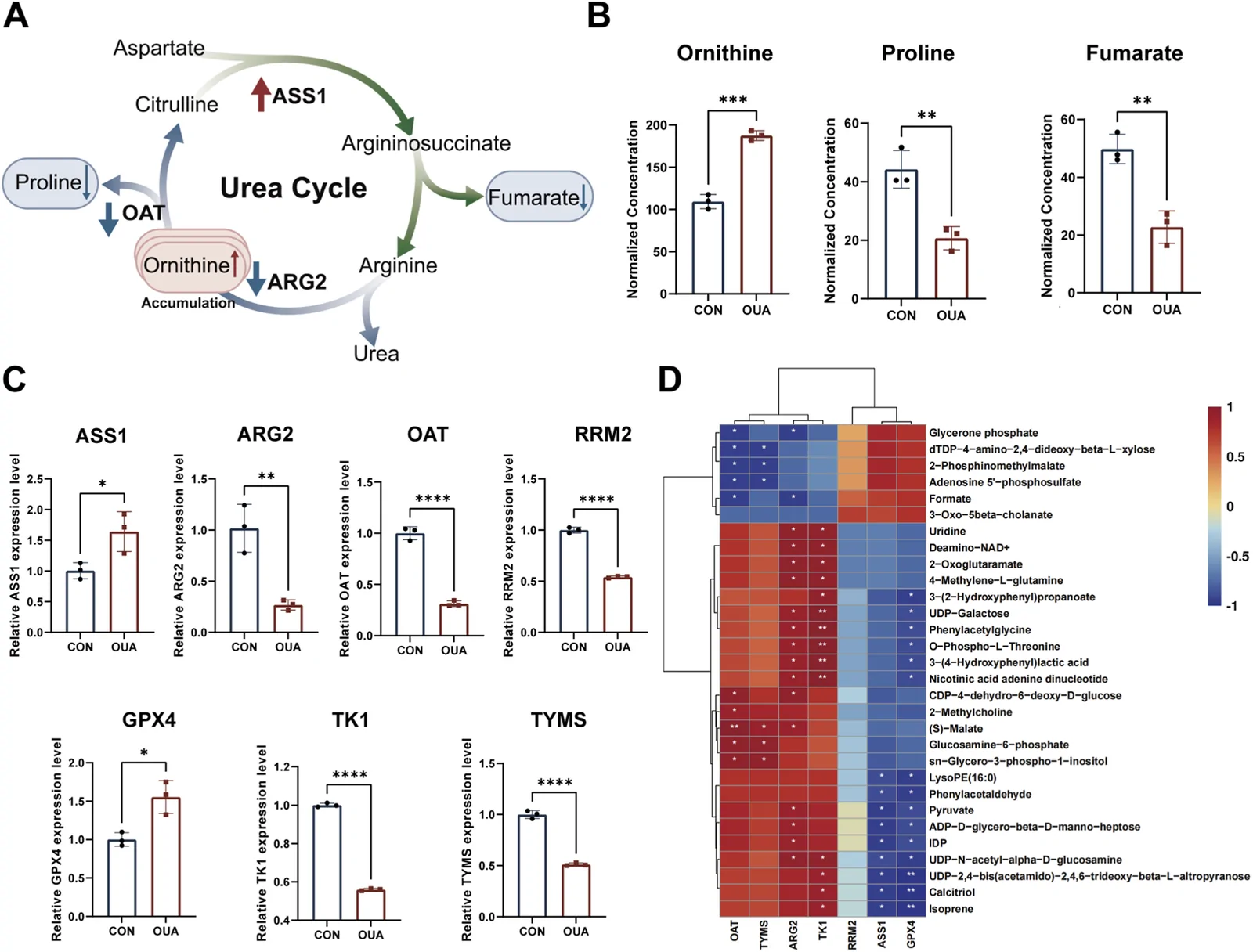
Ouabain reprograms the arginine-dependent metabolic network in DLBCL cells. **(A)** The KEGG pathway maps of Urea cycle (Blue indicates downregulation, red indicates upregulation.). **(B)** Targeted LC-MS/MS quantitative analysis of core metabolites (ornithine, proline, and fumarate). The absolute concentrations of metabolites were normalized to the total protein content of each corresponding sample (detailed parameters are available in Supplementary Table S4). **(C)** RT-qPCR validation of the mRNA expression levels of genes encoding key differentially expressed proteins (DEPs), including *ASS1, ARG2, OAT, GPX4, RRM2*, *TK1*, and *TYMS*. **(D)** Correlation heatmap between selected DEPs and DAMs. Data are presented as mean ± SD (n = 3) (*p < 0.05, **p < 0.01, ***p < 0.001, ****p < 0.0001).

In line with the changes in arginine metabolism, ouabain induced significant dysregulation in glutathione (GSH) metabolism. GSH levels and related metabolites, including glutamate, cysteinylglycine, and pyroglutamic acid, were significantly reduced (Figure 4D), indicating sustained depletion of the intracellular antioxidant pool that increases oxidative stress. The disruption of glutathione metabolism is tightly linked to the altered arginine metabolism, as studies have shown that arginine influences GSH synthesis (Liang et al., 2018; Phang, 2019; Xia et al., 2025). The upregulation of glutathione peroxidase 4 (GPX4), a key enzyme in lipid peroxidation detoxification, suggests a compensatory response to the increased oxidative burden. However, the depletion of GSH, coupled with the downregulation of RRM2, further links redox imbalance to nucleotide metabolism (Zuo et al., 2024). This cascade of metabolic alterations enhances the susceptibility of U2932 cells to ferroptosis, a non-apoptotic form of cell death induced by oxidative stress.

Ouabain also led to pronounced inhibition of nucleotide metabolism. Metabolomic analysis revealed significant reductions in pyrimidine-related metabolites, including UMP, UTP, and uridine (Figure 4D), suggesting diminished availability of nucleotide precursors. Correspondingly, proteomic data showed downregulation of key enzymes involved in nucleotide biosynthesis and salvage pathways, such as RRM2 (Supplementary Figure S3A3), thymidine kinase 1 (TK1), and TYMS (Figure 4C). These changes impaired the production of deoxyribonucleotides, triggering replication stress and inhibiting DNA synthesis. The inhibition of nucleotide synthesis coincided with glutathione depletion, which is crucial for maintaining nucleotide precursor pools (Sengupta et al., 2019; Tarangelo et al., 2022; Mullen and Singh, 2023). This suggests that nucleotide biosynthesis and redox regulation are interdependent, and the disruption of one pathway exacerbates deficiencies in the other. This combination of metabolic constraints significantly reduces tumor cell proliferation and survival, while also sensitizing cells to ferroptosis. In summary, ouabain triggers a coordinated disruption of key metabolic networks, with arginine metabolism serving as the central hub linking glutathione depletion and nucleotide biosynthesis inhibition.

### 3.4 Ouabain significantly altered the arginine metabolism profile according to targeted metabolomics

Based on the above results, we performed targeted metabolomic analysis, and a representative chromatogram of amino acids is shown in Supplementary Figure S6. After quantitative analysis of the metabolites *via* LC-MS/MS, as shown in Figure 5B, in the OUA group, proline (p = 0.0058) and fumarate (p = 0.0035) significantly decreased and ornithine (p = 0.0002) increased. Additionally, to provide a comprehensive view of this network, we quantified other core metabolic intermediates, including arginine, citrulline, aspartic acid, glutamic acid, and uridine (Supplementary Figure S7). These interconnected metabolic alterations lead to increased oxidative stress, replication stress, and growth inhibition, which together contribute to the anti-tumor effects of ouabain. By modulating arginine metabolism, ouabain not only affects amino acid balance but also orchestrates broader changes in cellular redox state and nucleotide metabolism, enhancing its therapeutic potential in DLBCL cells.

Based on the above findings, we performed RT-qPCR analysis to validate seven protein-coding genes associated with the enriched pathways (Figure 5C). In OUA vs. CON, *ASS1* (p = 0.0344), *GPX4* (p = 0.0145) were increased, *ARG2* (p = 0.0056), *OAT* (p < 0.0001), *RRM2* (p < 0.0001), *TK1* (p < 0.0001) and *TYMS* (p < 0.0001) were significantly decreased. The results were consistent with the conclusions of the KEGG enrichment analysis, further supporting the reliability of the integrated analysis.

Subsequently, correlation analysis was performed between the seven selected differentially expressed proteins and all differential metabolites identified in the OUA group versus the CON group (Figure 5D). ARG2 and OAT exhibited significant positive correlations with multiple amino acid intermediates, such as (S)-malate and 4-methylene-L-glutamine. In contrast, ASS1 was negatively correlated with (S)-malate. TYMS and TK1, both core enzymes for DNA synthesis, showed broad positive correlations with nucleotide precursors and intermediates, including uridine and O-phospho-L-threonine. RRM2 displayed only weak, non-significant correlations with most metabolites, implying that its regulatory mechanism may be more complex. GPX4 was strongly negatively correlated with nearly all listed metabolites, particularly calcitriol and isoprene, reflecting a pronounced depletion of various metabolites related to lipid, vitamin, and carbohydrate metabolism under an enhanced antioxidant state.

## 4 Discussion

DLBCL is the most prevalent subtype of B-cell non-Hodgkin’s lymphoma, up to 40% of cases still progress to refractory or relapsed disease, posing a formidable clinical challenge (Fei et al., 2022). In recent years, studies have reported that ouabain can promote apoptosis in various tumor cells, especially in DLBCL. This study conducted a systematical analysis of proteomics and metabolomics for the first time to reveal the multi-level mechanism by which ouabain exerts its anti-tumor effect in DLBCL cells and suggest that ouabain targets multiple metabolic networks to suppress tumor growth. The findings contribute to a deeper understanding of the mechanism of ouabain on DLBCL cells and provide a reference for the exploration of its combined treatment regimens.

We focused on differentially expressed proteins and metabolites between the CON and OUA groups in U2932 cells. Proteomic analysis revealed significant upregulation of proteins associated with immune recognition and stress responses, such as HLA-B polymorphisms, suggesting that ouabain may enhance immune surveillance of tumor cells (Alcoceba et al., 2013). Concurrently, key proteins involved in cell proliferation and DNA repair, including the critical oncogene BCL6(Schmitz et al., 2018), were significantly downregulated, indicating that ouabain targets multiple oncogenic pathways simultaneously. Through metabolomics studies, we can gain a deeper understanding of the changes in metabolites within DLBCL cells after administration of ouabain, which can help reveal the molecular mechanism and potentially identify new therapeutic targets. Squalene (Xu et al., 2020), a precursor of cholesterol synthesis, was significantly upregulated, suggesting that ouabain may block downstream steps of cholesterol synthesis, impairing the ability of tumor cells to invade and metastasiz. Conversely, taurine (Baliou et al., 2021), an important antioxidant, was significantly downregulated, potentially weakening the cellular resistance to oxidative stress, thus enhancing ouabain’s ability to induce oxidative damage. Moreover, the downregulation of nucleotide derivatives, such as UTP, suggests impaired DNA and RNA replication, further suppressing cell proliferation.

Particularly noteworthy, ouabain treatment resulted in a significant accumulation of intracellular ornithine levels, alongside reductions in proline and fumarate. Our subsequent analysis primarily focuses on the arginine-dependent network. However, these diverse alterations across lipids, amino acids, and nucleotides indicate that ouabain induces a broader metabolic reprogramming in DLBCL cells, potentially reflecting secondary adaptive responses or off-target effects. These changes may synergistically suppress DLBCL progression by destabilizing the tumor microenvironment and altering cellular energy metabolism. KEGG pathway analysis confirmed that ouabain simultaneously modulates multiple pathways, including cell cycle regulation, DNA repair, amino acid metabolism, energy metabolism, and oxidative stress, thereby systematically elucidating the molecular mechanism by which ouabain exerts its multi-target, multi-pathway antitumor effects in DLBCL.

Among these metabolic alterations, amino acid metabolism has emerged as a key regulatory axis. In particular, our integrated proteomic and metabolomic analyses revealed that arginine metabolism plays a central role in ouabain-induced metabolic reprogramming in DLBCL cells. Our data show that ouabain induces significant alterations in arginine metabolism, which emerges as a central hub linking multiple metabolic pathways. The upregulation of ASS1 and downregulation of ARG2 suggest that ouabain enhances the flux of arginine toward argininosuccinate while impairing its conversion to ornithine and urea. Multiple studies have confirmed (Wolfson and Sabatini, 2017; Casero et al., 2018; De Santo et al., 2018; Mossmann et al., 2018; Zou et al., 2019) that tumor cells are highly dependent on arginine. Importantly, a very recent study (Hong et al., 2025) reported that ouabain affects arginine metabolism in DLBCL through the IGF2BP2-GLS/NOS3 axis. While their work provides crucial insights into a specific upstream regulatory mechanism, our study extends this paradigm by providing a comprehensive, system-level landscape. Our data demonstrate that this arginine dysregulation does not occur in isolation; rather, it acts as a central node that links bioenergetic supply, redox homeostasis, and macromolecular biosynthesis. Consistent with the concept that arginine metabolism is closely intertwined with glutamine metabolism to regulate cellular oxidative stress and nucleotide synthesis (Liang et al., 2018; Phang, 2019; Mossmann et al., 2023; Xia et al., 2025), our network analysis reveals that the ouabain-induced arginine shift cascades into widespread downstream consequences. This holistic multi-omics view complements existing mechanistic insights by mapping the broader metabolic vulnerabilities, logically paving the way for the subsequent observations of redox imbalance and nucleotide suppression.

A key finding of this study is the disruption of redox homeostasis induced by ouabain. We observed a significant reduction in GSH levels, which is indicative of compromised cellular antioxidant capacity (Bansal and Simon, 2018). As a classic Na^+^/K^+^-ATPase inhibitor (Li et al., 2010), ouabain can directlyinduce elevated levels of ROS. However, its specific mechanism affecting ferroptosis in DLBCL remains unclear. GSH depletion, along with the concurrent upregulation of GPX4, points to increased oxidative stress within the cells and a hallmark of metabolic dysregulation in cancer cells (Stockwell et al., 2017; Bansal and Simon, 2018). Interestingly, our findings suggest that the changes in arginine metabolism may directly influence glutathione metabolism. Arginine serves not only as a critical precursor for glutamate synthesis, but also regulates GSH synthesis through the Nrf2 pathway (Liang et al., 2018). This functional link implies that the shift in arginine metabolic flux triggered by ouabain may limit the availability of key substrates required for GSH biosynthesis, thereby exacerbating GSH depletion and redox imbalance. The redox imbalance resulting from GSH depletion likely contributes to the sensitization of cells to ferroptosis, a form of cell death driven by lipid peroxidation and oxidative damage (Yu et al., 2023). By enhancing oxidative stress and depleting antioxidants, ouabain may exploit the vulnerabilities of DLBCL cells to ferroptosis (Schmitt et al., 2021), thus suggesting a potential novel mechanism for its antitumor activity.

In addition to its effects on redox regulation, ouabain significantly inhibited nucleotide biosynthesis, particularly pyrimidine metabolism, which is essential for DNA and RNA synthesis. We observed a marked reduction in UMP, UTP, and uridine, which are key precursors for nucleic acid synthesis. Correspondingly, proteomic analysis revealed the downregulation of enzymes such as RRM2, TK1, and TYMS, all of which play crucial roles in nucleotide production.

The suppression of nucleotide metabolism is consistent with the idea that rapidly proliferating tumor cells have a heightened demand for nucleotides to sustain DNA replication and repair (Turgeon et al., 2018; Liao et al., 2022). By disrupting this process, ouabain limits the availability of deoxyribonucleotides, which results in replication stress and cell cycle arrest, ultimately inhibiting tumor growth. Moreover, our data suggest that the impairment in nucleotide biosynthesis is linked to redox imbalance, as reduced GSH levels are known to influence nucleotide synthesis through the regulation of key enzymes like RRM2 (Sengupta et al., 2019; Tarangelo et al., 2022; Mullen and Singh, 2023). The downregulation of RRM2 not only directly impedes DNA replication and induces S-phase arrest but also potentially enhances cellular sensitivity to ferroptosis by reducing the dNTP pool (Zuo et al., 2024).

The integration of proteomic and metabolomic analyses in this study provides novel insights into how ouabain mediates metabolic reprogramming in DLBCL cells. By centrally targeting arginine metabolism, ouabain coordinates multiple metabolic pathways to inhibit tumor cell proliferation. This suggests its potential as a therapeutic agent in DLBCL. The concurrent disruption of redox regulation and nucleotide biosynthesis not only suppresses cell growth but also potentially enhances ferroptosis sensitivity, offering a multi-targeted approach to overcome the metabolic plasticity of lymphoma cells. Importantly, the reprogramming of metabolic networks by ouabain underscores the importance of metabolic vulnerabilities in cancer. Targeting these vulnerabilities, particularly through arginine metabolism, could provide a new avenue for therapeutic intervention in lymphoma and other metabolically dysregulated malignancies.

Although our study provides significant insights, several limitations should be addressed in future research. First, our current findings are based on a single DLBCL cell line (U2932), which represents the ABC subtype. It is necessary to validate these results in additional cell lines, particularly GCB-type models, to confirm the broader applicability of ouabain’s effects. Second, while our multi-omics signatures strongly suggest the involvement of ferroptosis, direct experimental evidence is currently lacking. Future investigations should include lipid peroxidation measurements and rescue experiments with ferroptosis inhibitors like Ferrostatin-1. Furthermore, the direct mechanistic link between ouabain’s classical target (Na+/K + -ATPase) and downstream arginine metabolism requires further elucidation. Finally, future studies should explore *in vivo* validation in mouse models. Combining ouabain with ferroptosis inducers, such as Erastin or RSL3, or with arginine-deprivation strategies could also be explored to enhance therapeutic efficacy in relapsed or refractory DLBCL.

## 5 Conclusion

In this study, we used integrative proteomic and metabolomic approaches to investigate the underlying mechanisms by which ouabain inhibits the proliferation of DLBCL. Our findings reveal that ouabain exerts its anti-tumor effects by centrally modulating arginine metabolism, which subsequently disrupts interconnected pathways, including redox regulation and nucleotide biosynthesis. We observed significant metabolic reprogramming following ouabain treatment, characterized by an accumulation trend of ornithine, decreased levels of proline and fumarate, glutathione depletion, and impaired nucleotide synthesis. These shifts are driven directly by the upregulation of ASS1 and downregulation of ARG2 and OAT, key enzymes governing arginine metabolic flux. Furthermore, RRM2 and GPX4 synergistically amplify oxidative stress and proliferation inhibition. Together, these findings provide a clearer understanding of the arginine-centered metabolic network targeted by ouabain. They highlight how metabolite depletion and oxidative stress synergistically exacerbate survival pressure on DLBCL cells. Further studies will explore specific molecules within this network as potential targets for combination therapies with ouabain, aiming to provide more tailored treatment options for patients with relapsed or refractory DLBCL.

## Data Availability

The mass spectrometry proteomics data have been deposited to the ProteomeXchange Consortium (https://proteomecentral.proteomexchange.org/) via the iProX partner repository with the dataset identifier PXD073963. The metabolomics data generated in this study have been deposited to the EMBL-EBI MetaboLights database (https://www.ebi.ac.uk/metabolights/) with the identifier MTBLS13870.
