# Supplementary material for: Integrative proteomic and metabolomic analysis reveals that ouabain inhibits DLBCL proliferation by reprogramming arginine-driven metabolic networks
Source: Front Pharmacol. 2026 Sep 21;17:1929830. doi: 10.3389/fphar.2026.1929830 (PMC13635548; doi:10.3389/fphar.2026.1929830)
Supplement: Supplementary file 1 [file DataSheet1.pdf]

Integrative Proteomic and Metabolomic Analysis Reveals that Ouabain Inhibits DLBCL Proliferation by Reprogramming Arginine-Driven Metabolic Networks

Yuan Wei[1†], Yanhuan Yu [2†], Yuning Zhu[1], Xinwen Zhang[1], Zhuoling Zhao[1], Sinan Wang[1], Fei Fei[1,2,3], Bei Cao[1,2,3], Runbin Sun[1,2,3*], Juan Li[1,2,3*]

[1]Department of Phase I Clinical Trials Unit, Nanjing Drum Tower Hospital Clinical College of Nanjing University of Chinese Medicine, Nanjing, China

[2]Department of Phase I Clinical Trials Unit, China Pharmaceutical University Nanjing Drum Tower Hospital, Nanjing, China

[3]Phase I Clinical Trials Unit, Nanjing Drum Tower Hospital, Affiliated Hospital of Medical School, Nanjing University, Nanjing, China

[†] Yuan Wei and Yanhuan Yu contributed equally to this work.

*Correspondence: Juan Li juanli2003@163.com Runbin Sun runbinsun@gmail.com

Supporting Information

The Supporting Information includes the following supplementary materials:

Figure S1: The viability of DLBCL cancer cell line treated with different concentrations of ouabain was measured via a Cell Counting Kit 8 assay (CCK-8).

Figure S2: Comparative group differential information bar chart.

Figure S3: Column plots showing DEPs related to ouabain.

Figure S4: OPLS-DA model permutation plots for OUA and CON groups.

Figure S5: Comparative group differential information bar chart.

Figure S6: Chromatograms and standard curves of amino acids.

Figure S7: Targeted quantification of core metabolic intermediates in DLBCL cells.

Table S1. List of abbreviations.

Table S2. Primer sequences used for RT-qPCR validation.

Table S3. Intersecting KEGG pathways and corresponding altered metabolites and proteins.

Table S4. Optimized MRM transitions and ion source parameters for targeted metabolites and internal standards.

Supplementary Data 2: List of proteins in CON and OUA groups. (xlsx)

Supplementary Data 3: List of metabolites in CON and OUA groups. (xlsx)

Supplementary Data 4: Peptide List. (xlsx)

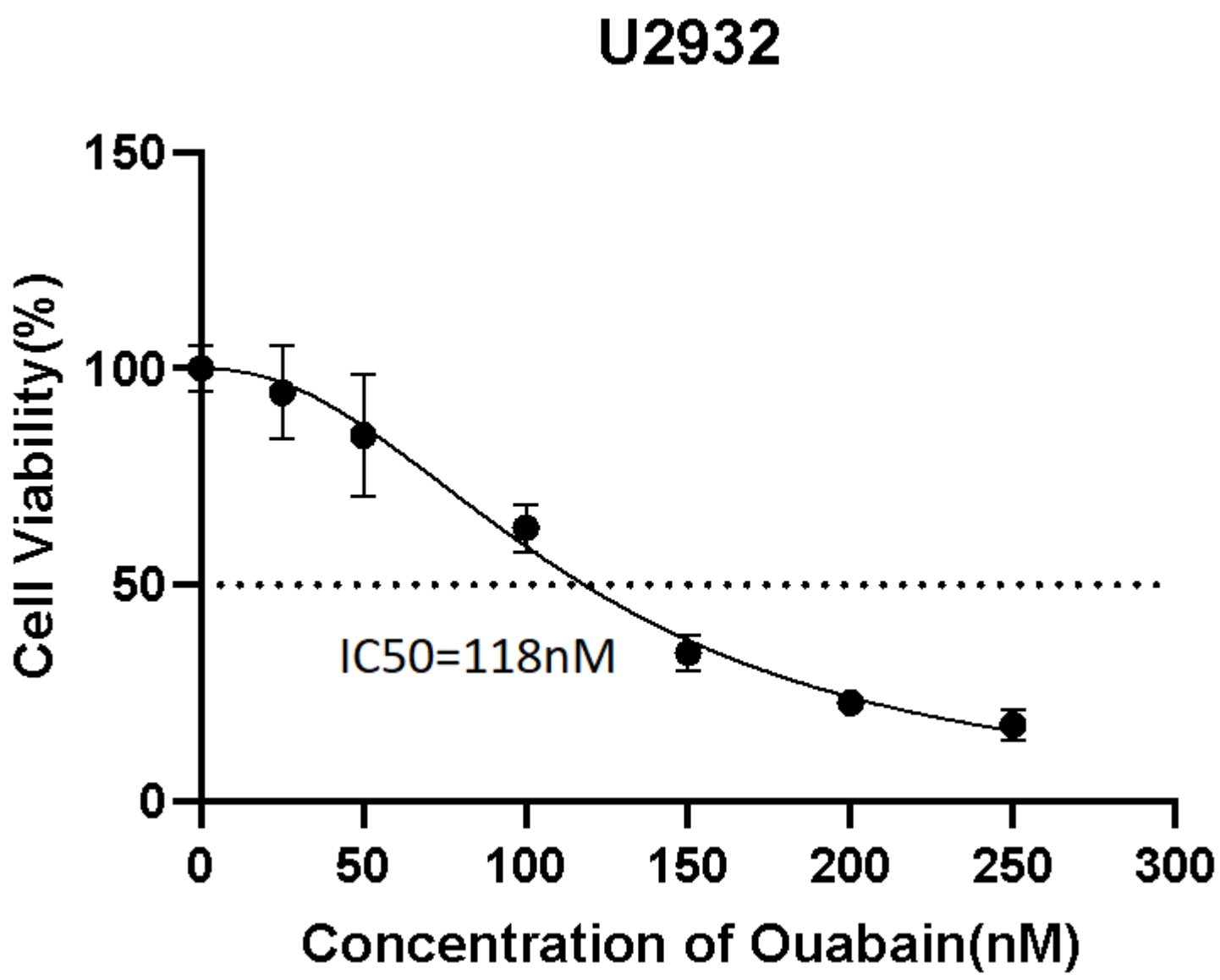

Figure S1. The viability of DLBCL cancer cell line treated with different concentrations of ouabain was measured via a Cell Counting Kit 8 assay (CCK-8). IC50, 50%

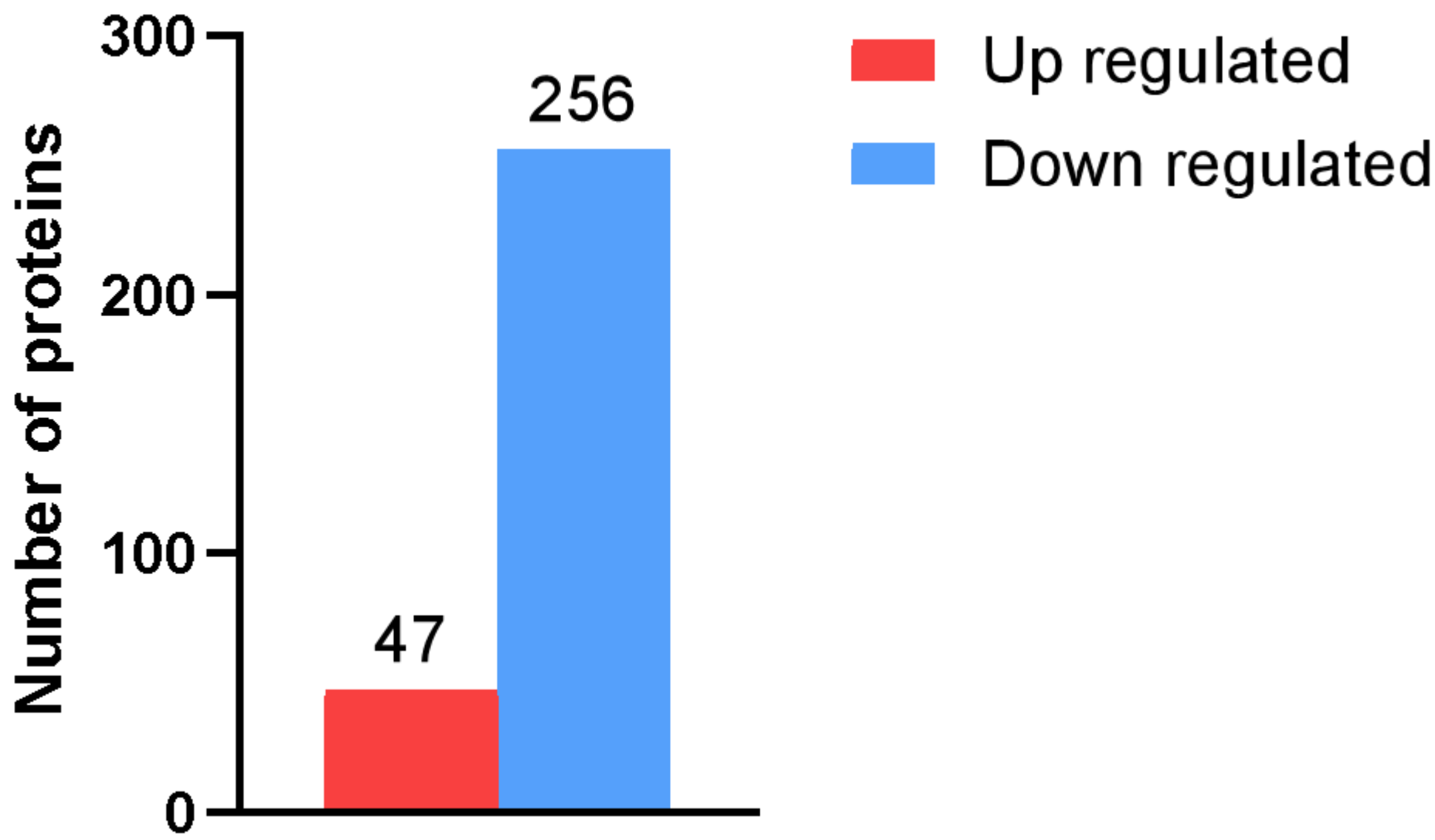

Figure S2. Comparative group differential information bar chart. Compared to the CON group, the OUA group has 47 upregulated DEPs and 256 downregulated DEPs.

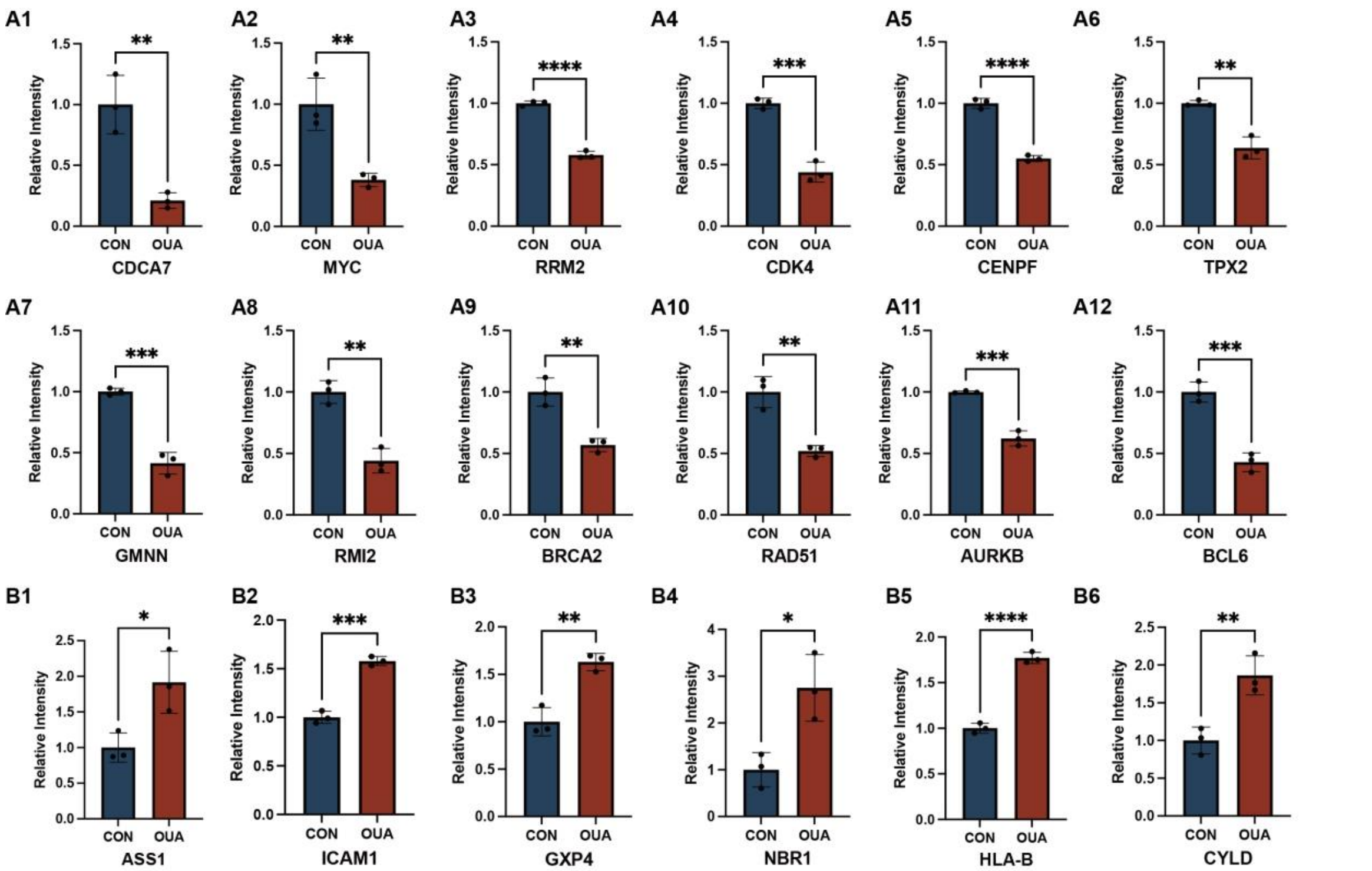

Figure S3. Column plots showing DEPs related to ouabain. The red and blue represent proteins that increased and decreased significantly between the CON and OUA groups. n = 3. *$p < 0.05$, **$p < 0.01$, ***$p < 0.001$, ****$p < 0.0001$ (Student's t-test).

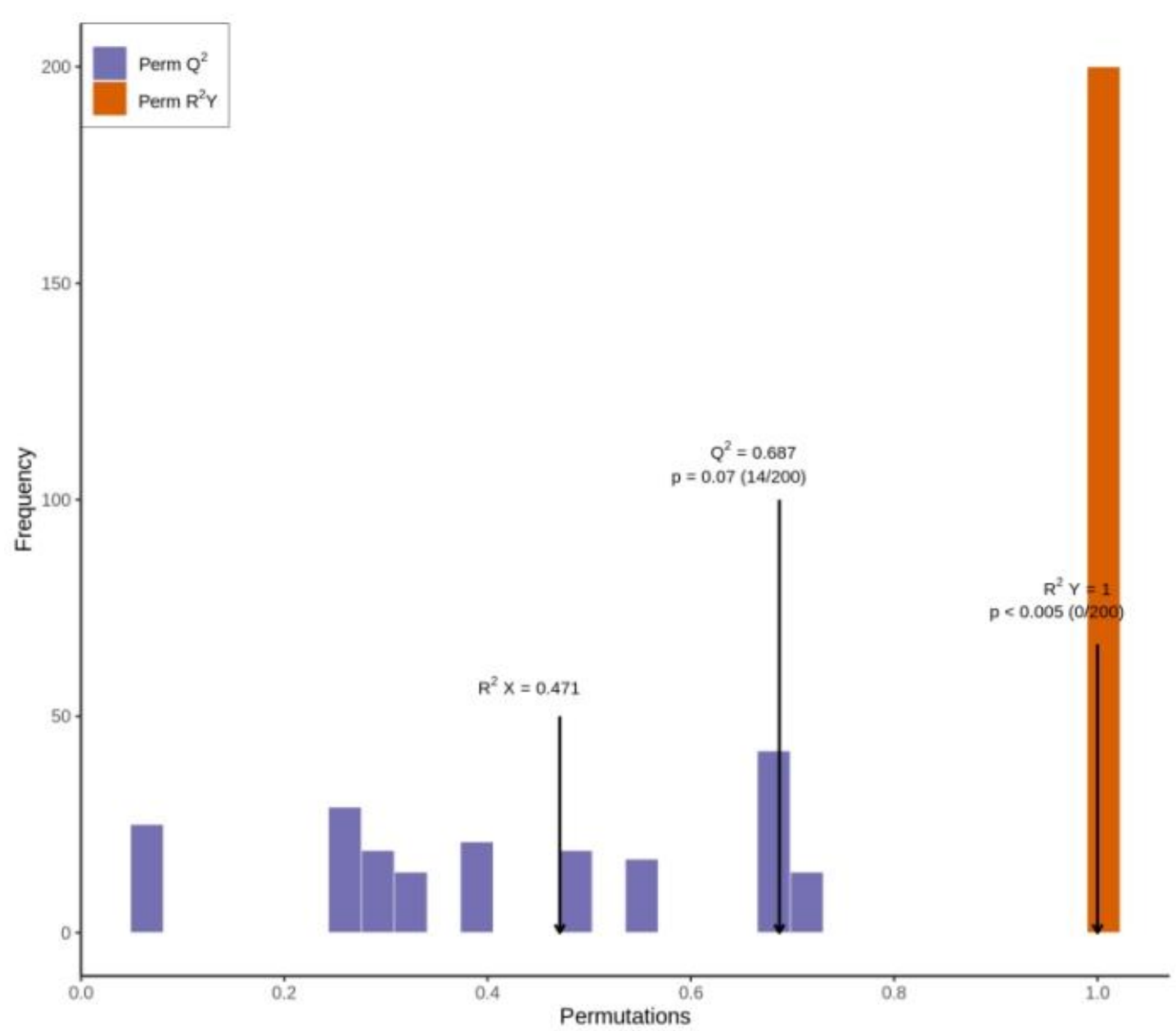

Figure S4. OPLS-DA model permutation plots for OUA and CON groups showing a high explanatory and predictive power for categorical variables.

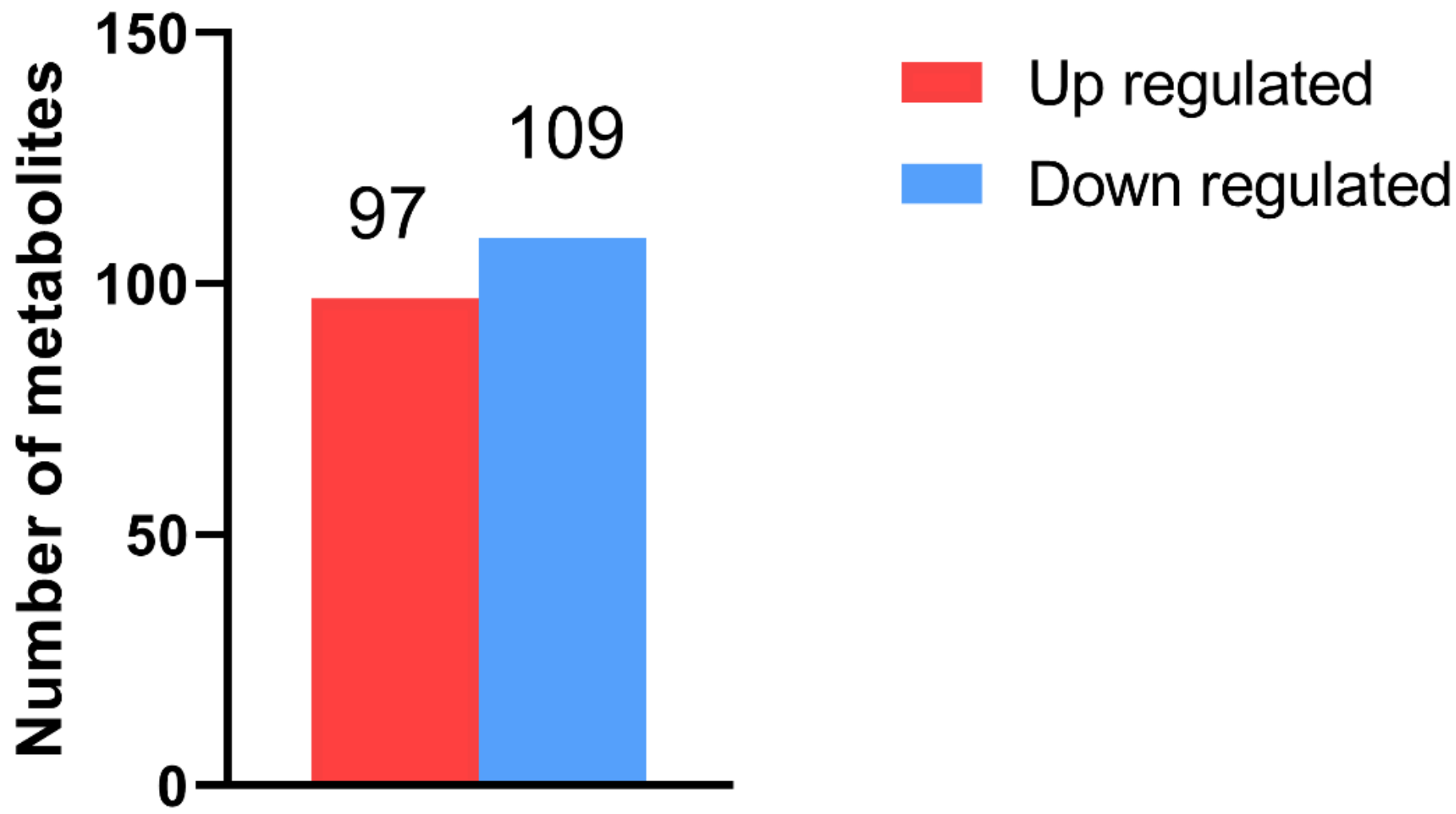

Figure S5. Comparative group differential information bar chart. Compared to the CON group, the OUA group has 97 upregulated DAMs and 109 downregulated DAMs.

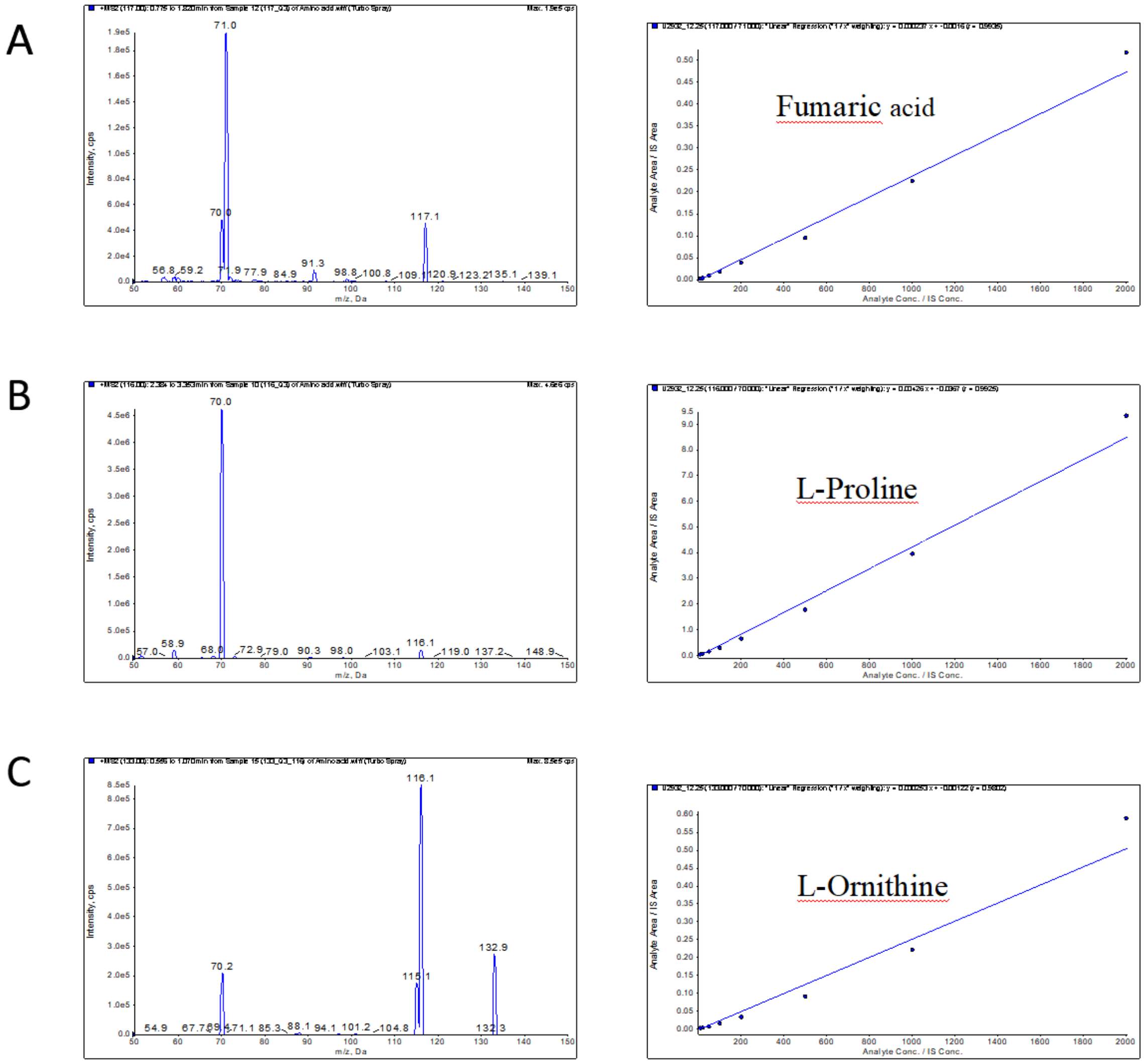

Figure S6. Chromatograms and standard curves of amino acids.

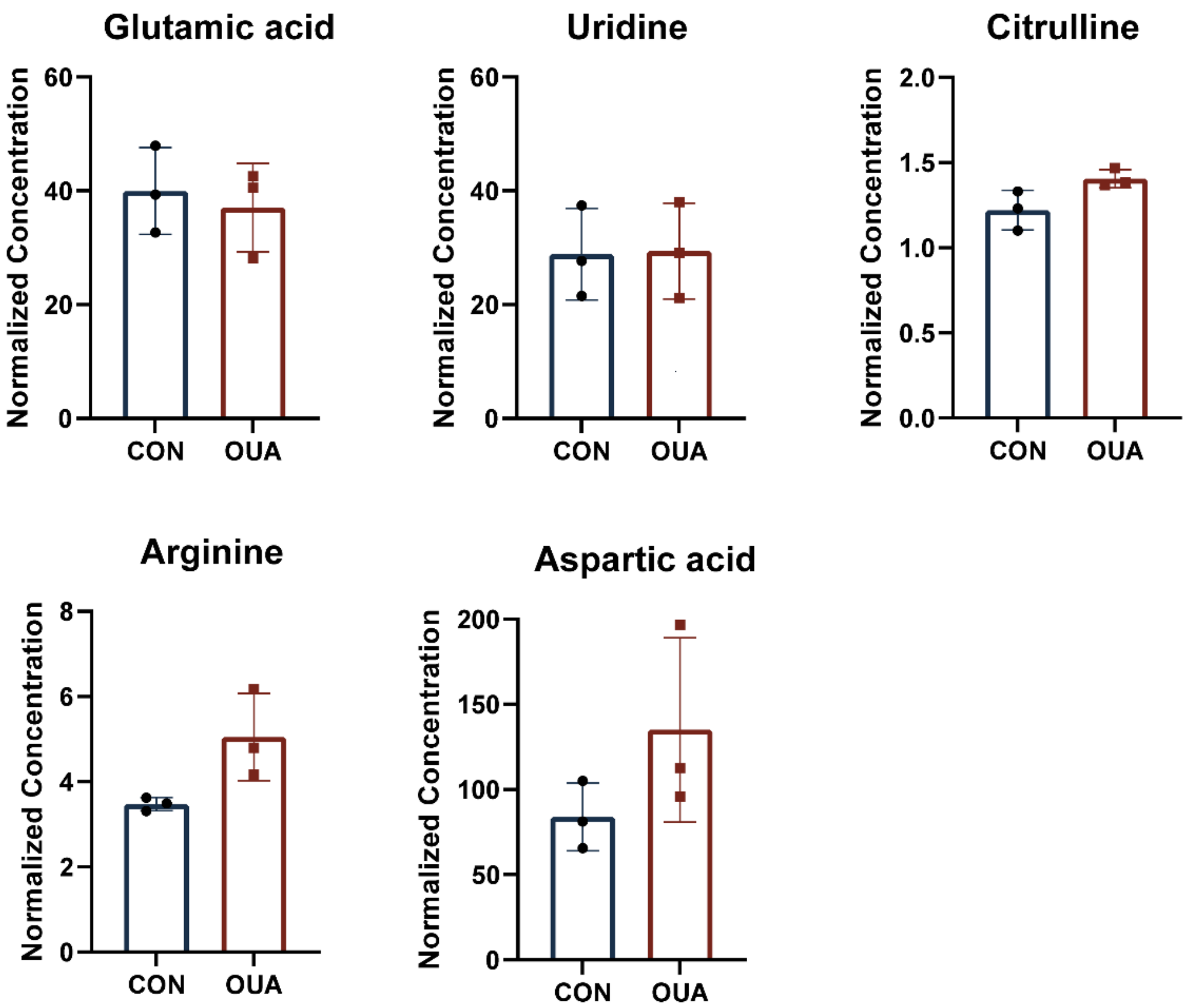

Figure S7. Targeted quantification of core metabolic intermediates in DLBCL cells.

Table S1. List of abbreviations.

Abbreviation	Complete name
ABC lymphoma	activated B-cell-like lymphoma
ASS1	Argininosuccinate synthase
AURKB	Aurora kinase B
BCL6	B-cell lymphoma 6 protein
BRCA	Breast cancer type 2 susceptibility protein
CDCA7	Cell division cycle-associated protein 7
CDK4	Cyclin-dependent kinase 4
CENPF	Centromere protein F
CYLD	Ubiquitin carboxyl-terminal hydrolase CYLD
CYP51A1	Lanosterol 14-alpha demethylase
DAMs	differentially abundant metabolites
DEPs	differentially expressed proteins
DLBCL	Diffuse large B-cell lymphoma
ESI−	electrospray ionization negative ion
ESI+	electrospray ionization positive ion
GCB lymphoma	B-cell-like lymphoma
GMNN	Geminin
GO	Gene Ontology
GPX4	Phospholipid hydroperoxide glutathione peroxidase
HLA-B	HLA class I histocompatibility antigen, B alpha chain
IC50	maximal inhibitory concentration
ICAM1	Intercellular adhesion molecule 1
KEGG	Kyoto Encyclopedia of Genes and Genomes
MCC	Maximal Clique Centrality
MNC	Maximum Neighborhood Component
MYC	Myc proto-oncogene protein
NHL	non-Hodgkin lymphoma
OAT	Ornithine aminotransferase, mitochondrial
PPI	protein-protein interaction
RAD51	DNA repair protein RAD51 homolog 1
RMI2	RecQ-mediated genome instability protein 2
ROS	Reactive oxygen species
RRM2	Ribonucleoside-diphosphate reductase subunit M2
SQLE	Squalene monooxygenase
TCA cycle	Citrate cycle
TK1	Thymidine kinase, cytosolic
TPX2	Targeting protein for Xklp2
TYMS	Thymidylate synthase

Table S2. Primer sequences used for RT-qPCR validation.

Primer names	Sequences (5’- 3’)
Actin forward	TGACGTGGACATCCGCAAAG
Actin reverse	CTGGAAGGTGGACAGCGAGG
ASS1 forward	GAGGATGCCTGAATTCTACA
ASS1 reverse	GTTGGTCACCTTCACAGG
ARG2 forward	ACCTGATAGTGAATCCACGCT
ARG2 reverse	CATGGGCATCAACCCAGAC
OAT forward	TTGCGCTTCATAGACGCC
OAT reverse	AAAACCACAGATCTGACAGCG
GPX4 forward	AGAGATCAAAGAGTTCGCCGC
GPX4 reverse	TCTTCATCCACTTCCACAGCG
RRM2 forward	CACGGAGCCGAAAACTAAAGC
RRM2 reverse	TCTGCCTTCTTATACATCTGCCA
TK1 forward	CCAAGTACCACAGCGATGAC
TK1 reverse	TGGACAGTCCTCACCAACG
TYMS forward	CTGCTGACAACCAAACGTGTG
TYMS reverse	GCATCCCAGATTTTCACTCCCTT
SQLE forward	CGCAGCGGTTACTCTGGTTA
SQLE reverse	AGCATCGCGCAGAACAACAC
CYP51A1 forward	GGCGATGGAGAAGGTGACAG
CYP51A1 reverse	TGGAGGACTTTTCACCCCTG

Table S3. Intersecting KEGG pathways and corresponding altered metabolites and proteins.

Pathway	p	DAMs / DEPs
Alanine, aspartate and glutamate metabolism	0.0001	Glutamate, 2-Oxoglutaramate, Fumarate, Pyruvate, Glucosamine-6P, beta-Citryl-L-glutamate
	0.3815	ASS1
Glutathione metabolism	0.0008	Glutathione, Glutamate, Cysteinylglycine, Pyroglutamic acid, Ornithine
	0.1760	GPX4/RRM2
Arginine biosynthesis	0.0060	Glutamate, Ornithine, Fumarate
	0.0349	ARG2/ASS1
Arginine and proline metabolism	0.0162	Proline, Glutamate, Ornithine, Pyruvate
	0.0263	ARG2/OAT/P4HA2
Pyrimidine metabolism	0.0213	UMP, UTP, Uridine, dUTP
	0.0385	RRM2/TK1/TYMS
Purine metabolism	0.0422	AMP, IDP, GMP, Guanosine, Adenylylsulfate
	0.8118	RRM2
Cysteine and methionine metabolism	0.2350	5'-Methylthioadenosine, Pyruvate
	0.4913	MTR
Glycerophospholipid metabolism	0.2660	Dihydroxyacetone phosphate; Choline
	0.7388	PLD2
Steroid biosynthesis	0.3190	Calcitriol, Squalene;
	4.33E-06	SC5D/CYP51A1/MSMO1/SQLE/FDFT1
Butanoate metabolism	0.3480	Glutamic acid
	0.2956	HMGCS1
Glycerolipid metabolism	0.3660	Dihydroxyacetone phosphate
	0.5706	PNPLA2
Metabolism of xenobiotics by cytochrome P450	0.5760	Benzo[a]pyrene-7,8-diol, 1,1-Dichloroethylene epoxide
	0.6424	EPHX1
Inositol phosphate metabolism	0.5760	Dihydroxyacetone phosphate
	0.0677	ITPK1/ITPKB/PIK3CG
Drug metabolism - other enzymes	0.6730	5-Fluorouridine diphosphate
	0.2802	RRM2/TK1

Table S4. Optimized MRM transitions and ion source parameters for targeted metabolites and internal standards.

Analyte	Q1 Mass	Q3 Mass	DP (volt)	EP (volt)	CE (volt)	CXP (volt)
Fumaric acid	117	71	100	10	18	10
L-Proline	116	70	107	10	18	8
L-Ornithine	133	116.1	91	10	24	8
4-Chlorophenylalanine (Internal Standard)	200.1	154	73	10	18.9	8
